# Association of inflammatory cytokines with mortality in peritoneal dialysis patients

**DOI:** 10.1051/bmdcn/2017070101

**Published:** 2017-03-03

**Authors:** Yao-Lung Liu, Jiung-Hsiun Liu, I-Kuan Wang, Shu-Woei Ju, Tung-Min Yu, I-Ru Chen, Yu-Ching Liu, Chung-Ming Huang, Shih-Yi Lin, Chiz-Tzung Chang, Chiu-Ching Huang

**Affiliations:** 1 Graduate Institute of Clinical Medical Science, China Medical University College of Medicine Taichung 404 Taiwan; 2 Department of Internal Medicine, China Medical University College of Medicine Taichung 404 Taiwan; 3 Division of Nephrology, China Medical University Hospital Taichung 404 Taiwan; 4 Division of Nephrology, Taichung Veteran General Hospital Taichung 407 Taiwan; 5 Department of Medical Research, China Medical University Hospital Taichung 404 Taiwan; 6 Division of Immunology and Rheumatology, China Medical University Hospital Taichung 404 Taiwan; 7 Graduate Institute of Integrated Medicine, China Medical University Taichung 404 Taiwan

**Keywords:** Proinflammatory cytokines, Peritoneal dialysis, Mortality

## Abstract

Aims: Previous study on association between pro-inflammatory cytokines and mortality in PD population is limited. We aimed to investigate here.

Methods: Total 50 patients who underwent incident PD were enrolled in this study. We measured the titers of pro-inflammatory cytokines Interleukin-18(IL-18), Interleukin-6 (IL-6), and Interleukin-1ß (IL-1ß). Study outcomes were all-cause mortality, cardiovascular-related mortality, and infection-caused mortality. Cox-regression model was used.

Results: In this 7 year prospective study, IL-18 ≥ 804.3pg/*ml*, IL-6 ≥ 3.92 pg/*ml*, IL-1ß ≥ 0.86pg/*ml*, age ≥ 50 years-old, and existence of diabetes could be used as individual significant predictors for mortality in PD patients. Higher titers of IL-6 were associated with lower averaging albumin levels within 1^st^ year of PD. Increasing numbers of these risk markers of mortality was associated with decreasing survival advantages (*P* = 0.001).

Conclusion: Age ≥ 50 years-old, diabetes, and inflammatory cytokines profiles at the start of PD therapy could predict for 7-year mortality in PD population.

## 1. Introduction

The prevalence of peritoneal dialysis (PD) is increasing in developing countries.[1] In 2008, there have been out of 197000 patients on PD. In terms of survival rate[2] and quality of life [3], PD and hemodialysis(HD) are usually recognized as clinical modality equivalents [4]. With the potential for cost saving, PD is widely promoted in developing countries, especially in Asia [5]. Many studies showed that PD is associated with a survival advantage that would diminish over time, while the causes are heterogeneous and still under investigation [6–8]. Given PD is often suggested for young non-comorbid population, it would be of considerable interest to investigate if any predictors of mortality available for patients at the inception of PD.

Uremic inflammation, characterized by increasing expression of pro-inflammatory cytokines, is a common status among patients with renal function impairment [9]. It is believed that these alterably activated cytokines and dys-regulated acute-phase reactants could predispose uremic patients to immunosuppressive status, cancer, malnutrition, and mortality [10–13].

Previous studies have used single inflammatory marker such as C-reactive protein or interleukin-6 (IL-6) to predict mortality in HD patients [14–19]. Few studies have investigated the association between pro-inflammatory cytokines and mortality in PD patients. Crosslinks among malnutrition, inflammatory cytokines, age, residual renal function, and mortality are still investigated [20–22]. Given current trend of recommending PD as first dialysis modality in developing countries, to investigate the predictors of mortality is worth for PD population.

Thus, we conducted a 7-year prospective study by first assessing the association among1) the pro-inflammatory cytokines Interleukin-18(IL-18), IL-6, and Interleukin-1ß(IL-1ß) and allcause mortality; 2) these cytokines, age, comorbidities, and malnutrition to find out the mortality predictors for PD patients.

## 2. Materials and methods

### 2.1. Study design and patients

During the period of November 2007 to December 2008 at China Medical University Hospital in Taiwan, a total of 50 patients with end-stage renal disease and who underwent incident PD were selected for this study. Only patients who fulfilled the following inclusion criteria were considered for enrollment. (i) patients who had not received other renal replacement therapy and for whom PD was the initial therapy type at the study baseline; (ii) patients who had been clinically stable for three months before entry, without infection or other active diseases; (iii) patients who had not had a documented chronic inflammation disorder or other malignant disease; and (iv) patients who did not take any immunosuppressants. The dialysis regimen and frequency of each patient were prescribed by their attending nephrologist. Clinical indications were the main reasons for any changes in dialysis regimen and frequency. The study was approved by and institutional review board at China Medical University Hospital and all enrolled patients had written informed consent.

### 2.2. Data collection

Clinical information was obtained by hospital records and dialysis logs. Blood samples were drawn in the morning after overnight fasting. Demographic information was included as confounders at the study baseline. Diabetes was defined *via* a previous physician’s diagnosis, or a fasting glucose of more than 126 mg/*dl* (7.0 mmol/L), or a non-fasting glucose of more than 200 mg/*dl* (11.1 mmol/L) at baseline. Weekly creatinine clearance (WCrCl; liters per week), Kt/V (urea clearance (K) multiplied by dialysis time (t) divided by the distribution volume (V)), urea, and a peritoneal equilibrium test were performed using a 2.27% dextrose solution for a 2-L volume exchange instilled in the peritoneal cavity within three months of initial dialysis. On the day of investigation, a 4 hour-collection of the dialysis fluid and urine was gathered to calculate dialysis adequacy. The ratio of creatinine concentration in the dialysate to the plasma (D/P creatinine) was reported. The Kt/V and WCrCl values in this study were the summary of residual renal and peritoneal clearances. Plasma IL-18, IL-6, and IL-1ß cytokine concentrations were determined by ELISA with kits from BioSource International (Camarillo, CA, USA)

### 2.3. Endpoint and outcome analyses

Patient mortality and causes of mortality are interests of investigation in this study. We followed up these patients until transferred to alternative renal replacement therapies (hemodialysis and kidney transplantation), lost to follow up, or ended on 31 Dec 2014.

### 2.4. Statistical analysis

Categorical variables were expressed as absolute numbers or percentages. Categorical data were compared by a, χ^2^-test if the observation numbers in all categories were larger than 5; otherwise, Fisher’s exact test was used. Continuous data were expressed as mean and the standard deviation (SD). Multivariate Cox proportional hazards regression was used to assess the independent predictors of death. Comparisons between the survivor and mortality groups were performed using the non-parametric Mann-Whitney U-test. The receiver-operator curve analysis and Youden’s index were used to determine the best cut-off point, one which maximized the sum of sensitivity and specificity for predicting mortality. Survival curves were generated using the Kaplan-Meier technique and tested using the log-rank test. All p-values were two sided, and a *p* < 0.05 was considered significant. All calculations were performed with the SPSS software version 20.0 for Windows (SPSS Institute, Chicago, IL).

## 3. Results

### 3.1. Patient characteristics

From November 2007 to December 2008, we identified 50 patients (21 male and 29 female) with ESRD and who underwent incident PD. The underlying kidney disease of these 50 patients was as follows. 20 patients had DM nephropathy (40%); 16 patients with chronic glomerulonephritis (32%); 5 patients with hypertensive nephrosclerosis (10%); 5 patients with chronic interstitial nephritis (10%); and 4 patients with other miscellaneous causes (8%). The 84-month overall mortality rate was 48% in the patients of the study. Table 1 demonstrates characteristics of patients in the survivor and mortality groups at the study’s baseline. The survivor group is younger than the mortality group. (50.42 ± 12.63 *vs.* 62.04 ± 13.4, *p* = 0.003) There are more diabetic patients in the mortality group (*p* < 0.005). The other characteristics including WBC counts, levels of hemoglobin, the normalized protein catabolic rate (nPCR), levels of serum calcium, serum phosphate, alkaline phosphatase, an intact parathyroid hormone (iPTH), WCrCl, peritoneal solute transport rate, and Total Kt/V urea revealed no significant difference between the survivor and mortality groups.

**Table 1:** Clinical characteristic of the study subjects at study baseline.

	Survivors (n = 26)	Death (n = 24)	*P* value
Age (yrs)	50.42 ± 12.63	62.04 ± 13.4	0.003
Men (%)	13 (50%)	8 (33.3%)	0.233
BMI (kg/m^2^)	23.64 ± 3.59	22.92 ± 3.23	0.462
Diabetes (%)	4 (15.3%)	16 (66.7%)	<0.005
Hemoglobin (g/*dl*)	9.96 ± 1.46	9.96 ± 1.59	0.998
Albumin at stating PD (gm/*dl*)	3.61 ± 0.40	3.37 ± 0.4	0.045
Albumin averaged within 1^st^ year of PD (gm/*dl*)	3.69 ± 0.32	3.16 ± 1.03	0.018
Calcium (mg/*dl*)	8.98 ± 0.60	8.93 ± 0.90	0.493
Phosphate (mg/*dl*)	5.29 ± 1.04	4.92 ± 1.46	0.316
Alkaline phosphatase	65.8 ± 32.1	81.2 ± 29.2	0.083
Intact PTH	390.8 ± 386.9	214.7 ± 202.1	0.052
nPCR	1.11 ± 0.25	1.02 ± 0.24	0.259
Transporters (H. HA. L. LA)	2.15.8.1	2.7.12.3	0.423
Total WCrCl (L/week/1.73m^2^)	75.14 ± 25.96	75.82 ± 23.41	0.922
Total Kt/V urea	2.06 ± 0.53	2.06 ± 0.4	0.997
Urine CCr	34.54 ± 26.27	37.97 ± 23.00	0.627
Urine Kt/V urea	0.7 ± 0.51	0.7 ± 0.37	0.977

BMI = Body mass index; Transporters. H. high, HA. high average, L. low, LA. low average; WCrCl = weekly creatinine clearance; K/t/V = urea clearance normalize to its volume distribution. PTH = parathyroid hormone; nPCR = normalized protein catabolic rate.

### 3.2. Pro-inflammation cytokines and all-cause mortality

**Fig. 1:**
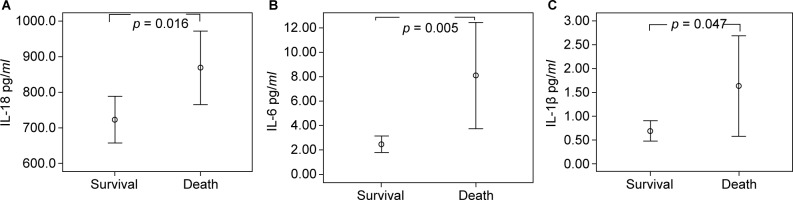
Levels of IL-18, IL-16, and IL-1ß between survival group and death group.

Of the 24 patient deaths during the course of the study, the causes of death were cardiovascular disease (CVD) in 6 patients, peritonitis in 7 patients, non-peritonitis infections in 9 patients, GI bleeding in 1 patient, and malignancy in 1 patient. We compared the baseline values of IL-18, IL-6 and IL-1ß between the survivor and mortality groups. Fig. 1 shows that the mortality group had higher IL-18 titers than the survivor group (867.91 pg/*ml* ± 231.51 pg/*ml* *vs.* 723.32 ± 153.97 pg/*ml*, *p* = 0.016). The mortality group had higher IL-6 titers than the survivor group (8.09 ± 8.97 pg/*ml* *vs.* 2.47 ± 1.56 pg/*ml*, *p* = 0.005). The IL-1ß titer is significantly higher in mortality group (1.44 ± 1.19 pg/*ml* *vs.* 0.74 ± 0.34 pg/*ml*, *p* = 0.047).

On the basis of the receiver-operator curve, the most appropriate cut-off point for IL-18 to predict all-cause mortality was 804.3 pg/*ml* (sensitivity 59.0%; specificity 67%; area under the curve = 0.629). The cut-off point for IL-6 to predict all-cause mortality was 3.92 pg/*ml* (sensitivity 63%; specificity 92%; area under the curve = 0.77). The cut-off point for IL-1ß to predict allcause mortality was 0.86 pg/*ml* (sensitivity 70%; specificity 80%; area under the curve = 0.75).

**Fig. 2:**
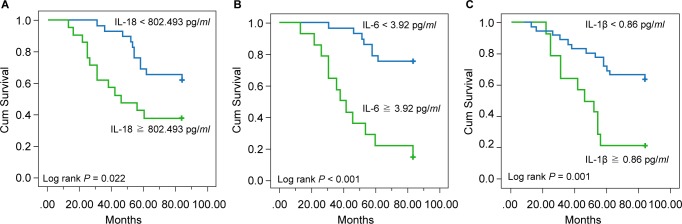
Survival curve of groups defined by levels of pro-inflammatory cytokines (A) IL-l8 with cut-off value of 804.3 pg/*ml*. (B) IL-6 with cut-off value of 3.92 pg/*ml*. (C) IL-lß with cut-off value of 0.86 pg/*ml*.

The Kaplan-Meier representation of the patient survival rate is shown in Fig. 2. The overall 84-month survival rate differed significantly among patients whose baseline titer of IL-18, IL-6, and IL-1ß were more or less than their corresponding cut-off points in each comparison (*p* = 0.022 in IL-18 comparison, *p* < 0.001 in IL-6 comparison, and *p* = 0.001 in IL-1ß comparison).

### 3.3. Relationship between pro-inflammation cytokines, cardiovascular-mortality, and infection-mortality

**Fig. 3:**
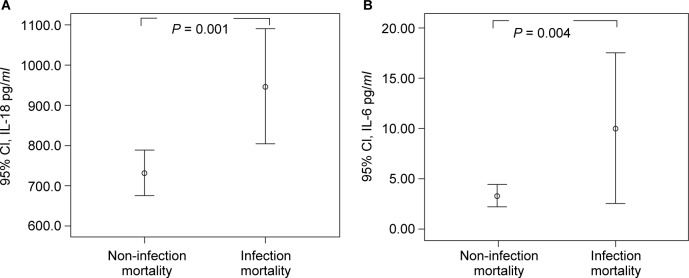
Levels of IL-18 and IL-16 between non-infection mortality group and infection-mortality group.

We further determined the association between pro-inflammatory cytokines and causes of mortality. A higher level of IL-18 is related to infection mortality (*p* = 0.001). The mean titer of IL-18 of the infection mortality group was higher than the non-infection mortality group (Fig. 3A, 947.92 ± 236.56 pg/*ml vs.* 731.23 ± 158.24 pg/*ml*, *p* = 0.001). The mean titer of IL-6 of the infection mortality group was higher than the non-infection mortality group (Fig. 3B, 9.88 ± 10.99 pg/*ml* *vs.* 3.28 ± 3.01 pg/*ml*, *p* = 0.004). Although there seems to be an association between titers of IL-1ß and infection mortality, the difference is insignificant (*p* = 0.052). None of the levels of IL-18, IL-6, nor IL-1ß are related to CV-cause mortality (*p* = 0.963 for IL-18, *p* = 0.842 for IL-6, and *p* = 0.641 for IL-1ß, respectively).

To clarify whether there is a baseline bias between CV-mortality group and infection-mortality group, we compared survival lengths and titers of proinflammatory cytokines between the two groups. CV-mortality group and infection-mortality group had similar lengths of survival (44.67 ± 13.10 months *vs.* 38.27± 16.36 months, *p* = 0.405). CV-mortality group and infection-mortality group had similar titers of pro-inflammatory cytokines (IL-18.834.64 ± 185.18 pg/*ml* *vs.* 867.86 ± 247.34 pg/*ml*, *p* = 0.784; IL-6. 4.46 ± 3.74 pg/*ml* *vs.* 8.20 ± 10.21 pg/*ml*, *p* = 0.442; IL-1ß. 0.77 ± 0.52 pg/*ml* *vs.* 1.48 ± 1.25 pg/*ml*, *p* = 0.466, respectively).

## 4. Associations between pro-inflammatory cytokines and clinical parameters

Age, diabetes, albumin levels, and residual renal function are reported to be related to inflammatory status or mortality in dialysis population.[23, 24] Thus, we clarified these associations between age, diabetes, and levels of albumin, urine CCr, and urine KT/V with these pro-inflammatory cytokines in our study’s patients (table 1). In Table 2, the correlation matrix shows that there were tight links among levels of IL-6, lower levels of albumin at the start of PD, lower averaging levels of albumin within the 1^st^ year of PD, and the existence of diabetes.

**Table 2:** Correlation matrix of IL-18, IL-6,IL-1ß, Age, Diabetes, Albumin levels at the start of peritoneal dialysis, average of albumin levels within the first year of peritoneal dialysis, Urine Ccr, and Urine KT/V.

	IL-18	IL-6	IL-1ß	Age	Diabetes	Albumin (A)	Albumin (B)	Urine Ccr
IL-18	–	–	–	–	–	–	–	–
IL-6	0.15 (0.38)	–	–	–	–	–	–	–
IL-1ß	0.36 (0.10	0.22 (0.35)	–	–	–	–	–	–
Age	0.24 (0.11)	0.30 (0.06)	0.09 (0.68)	–	–	–	–	–
Diabetes	0.10 (0.50)	0.41 (0.007)	0.38 (0.07)	0.19 (0.18)	–	–	–	–
Albumin (A)	0.03 (0.85)	−0.35 (0.02)	0.01 (0.95)	−00.11 (0.44)	−00.42 (0.02)	–	–	–
Albumin (B)	−00.004 (0.98)	−00.47 (0.02)	−00.13 (0.55)	−00.29 (0.04)	−00.49 (<0.001)	0.769 (0.001)	–	–
Urine Ccr	−00.07 (0.67)	0.03 (0.87)	−00.29 (0.17)	0.18 (0.21)	0.08 (0.55)	0.08 (0.57)	0.16 (0.27)	–
Urine KT/V	−00.06 (0.70)	−00.04 (0.79)	−00.27 (0.21)	0.21 (0.15)	−00.09 (0.51)	0.17 (0.24)	0.28 (0.05)	0.89 (<0.001)

*Spearman rank correlation coefficients and*p* values.

Albumin (A). albumin levels at inception of PD.

Albumin (B). averaging albumin levels within the 1^st^ year of PD.

## 5. Predictive factors of all-cause mortality

In this study, age, diabetes, and levels of albumin were potential predictors of mortality. We searched for the most appropriate cutoff point of age and levels of albumin to predict mortality. The cut-off point for age was 50 years-old (sensitivity 88%; specificity 62%; area under the curve = 0.75). The best cut-off point for levels of albumin was 3.55 gm/*dl* (sensitivity 53%; specificity 71%; area under the curve = 0.66).

A Cox regression model was used to identify independent significant predictors of mortality among pro-inflammatory cytokines and clinical parameters. On completing an univariate Cox regression analysis, IL-18, IL-6, IL-1ß, an age ≥ 50 years-old, and diabetes were all significantly associated with all-cause mortality, whereas albumin levels < 3.55 gm/*dl* at the start of PD were found to not be an independent risk factor for all-cause mortality (*p* = 0.096) (Table 3).

**Table 3:** Univariate Cox regression model of IL-18, IL-6, IL-1ß, age > 50 years old, diabetes, albumin on survival function of peritoneal patients

	Odds ratio	*P* value
IL-18 ≧ 804.3 pg/*ml*	2.62	0.025
IL-6 ≧ 3.92 pg/*ml*	6.90	< 0.001
IL-1ß ≧ 0.86 pg/*ml*	5.46	0.01
Age ≧ 50 years-old	6.83	< 0.001
Diabetes Mellitus	4.73	< 0.001
Albumin (A)	2.77	0.096
Albumin (B)	6.88	0.022

Albumin (A). albumin levels at inception of PD.

Albumin (B). averaging albumin levels within the 1^st^ year of PD.

Though a lower albumin level at the start of PD could not predict mortality, we averaged serial levels of albumin within the 1^st^ year of PD. The mortality group had lower averaging albumin levels within the 1^st^ year of PD than the survivor group (3.16 ±1.03 gm/*dl* *vs.* 3.69 ± 0.32 gm/*dl*, *p* = 0.018). The cut-off point for averaging albumin levels to predict all-cause mortality was 3.8 pg/*ml* (sensitivity 42.3%; specificity 92%; area under the curve = 0.692). A Cox regression analysis showed that averaging albumin levels <3.8 gm/*dl* within the 1^st^ year of PD could be an independent risk factor for all-cause mortality (p=0.022) (Table 3).

## 6. All-cause mortality prediction model

**Fig. 4:**
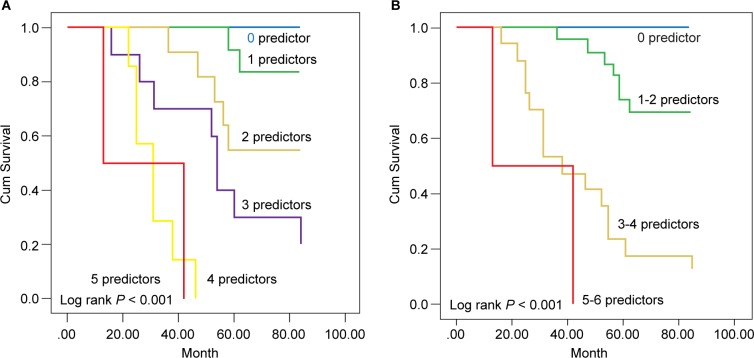
(A) Survival curves of groups with 0, 1, 2, 3, 4, or 5 predictors. Definition of predictors. existence of diabetes mellitus, age ≧ 50 years, IL-18 ≧ 804.3 pg/*ml*, IL-6 ≧ 3.92 pg/*ml*, and IL-1ß ≧ 0.86 pg/*ml*. (B) Survival curves of groups with 0, 1-2, 3-4, or 5-6 factors. Definition of factors, existence of diabetes mellitus, age ≧ 50 years, IL-18 ≧ 804.3 pg/*ml*, IL-6 ≧ 3.92 pg/*ml*, IL-1ß ≧ 0.86 pg/*ml*, and averaging albumin level < 3.8 gm/*dl* within the first year of PD.

To establish a risk prediction model, we defined 5 predictors for all-cause mortality. Diabetes mellitus, age ≧ 50 years, IL-18 ≧ 804.3 pg/*ml*, IL-6 ≧ 3.92 pg/*ml*, and IL-1ß ≧ 0.86 pg/*ml*. In Fig. 4a, the survival curve of each group of 0, 1, 2, 3, 4, or 5 predictors demonstrates a decreasing survival advantage with an increasing numbers of predictors (*p* < 0.001).

Another modified risk prediction model which incorporated the nutrition status in the first year of starting PD was also proposed. It included 6 markers. Diabetes mellitus, age ≧ 50 years, IL-18 ≧ 804.3 pg/*ml*, IL-6 ≧ 3.92 pg/*ml*, IL-1ß ≧ 0.86 pg/*ml*, and average levels of albumin <3.8 gm/*dl*. Fig. 4b shows the survival function of each group with 0, 1-2, 3-4, 5-6 markers, respectively (*p* < 0.001).

## 7. Discussion

The predictive association between major events and inflammatory cytokines including IL-6, and IL-18 are reported repeatedly in abundant studies, especially CKD and HD patients [14, 25–29]. Few studies have used a combination of these markers to predict major outcomes in PD patients. Wang *et al.* first used CRP. IL-6, Fetuin A, and valvular calcification as a composite. In their 4-year follow-up study, they assessed the predictive role of multi-inflammatory markers for CV events and all-cause mortality [19]. However, their PD patients were heterogeneous. The dialysis duration and residual renal function were significantly different among their study’s subgroups, of both had impact on mortality [30, 31]. Our results could provide a more prognostic value of multi-inflammatory markers of mortality for PD population. Our data was also complementary to the findings of Wang *et al.* since our study subjects were incident and no difference of residual renal function and duration of dialysis existed between the survivor and mortality group [19].

In our study, the plasma levels of IL-18, IL-6, and IL-1ß were associated with infection-cause mortality but not CV-cause mortality. Previous studies have showed that increasing levels of IL-18 and IL-6 have been found to be involved in the pathogenesis of atherosclerosis and associated with future CV events and mortality [32–34]. Since the survival lengths were similar between CV-cause mortality and infection-cause mortality in our study groups, we propose that the high inflammatory status could predispose a PD patient to either a CV event or an infection event equally. The possibility of competing mortality bias between a CV event and infection would be the cause for this discordance of our study with previous studies [27, 35].

In our study, we noted PD patients with low averaging levels of albumin within the 1^st^ year of PD had survival disadvantages. Higher Il-6 levels were tightly associated with low averaging levels of albumin within the 1^st^ year of PD. Since the initiation of PD, the gap of albumin levels between the survivor and mortality groups became wider with time despite initial similar values of nPCR between these two groups. Our results pointed out that single albumin data might not be as good predictors for mortality as averaging levels of albumin.

Age, diabetes, and higher levels of IL-6 were correlated with Month Month

Fig. 4 - (A) Survival curves of groups with 0, 1, 2, 3, 4, or 5 predictors. Definition of predictors. existence of diabetes mellitus, age 2: 50 years, IL-18 2 804.3 pg/*ml*, IL-6 2 3.92 pg/*ml*, and IL-1B 2 0.86 pg/*ml*. (B) Survival curves of groups with 0, 1-2, 3-4, or 5-6 factors. Definition of factors, existence of diabetes mellitus, age 2 50 years, IL-18 2 804.3 pg/*ml*, IL-6 2 3.92 pg/*ml*, IL-1B 2 0.86 pg/*ml*, and averaging albumin level < 3.8 gm/*dl* within the first year of PD.

malnutrition and morality in our PD patients, consistent with well-established findings that malnutrition and inflammation are synchronized in patients with renal failure [36]. The causes of increasing IL-6 levels in ESRD patients is multifaceted including decreased renal elimination of IL-6, increased production of IL-6 from oxidative stress, or from comorbidities such as congestive heart failure [37–39]. Previous studies have showed using statin, angiotensin converting enzyme inhibitors, fish oil, or aerobic exercise could lessen IL-6 levels in renal patients [40-43]. Future studies might be needed to investigate the effects of life style modification or anti-cytokine therapy could provide survival benefit for PD population.

Some limitations need to be mentioned in this study. First, the size of this group study was small. However, these patients were carefully evaluated and followed up for 7 years, which could provide adequate information and compensate for this limitation. Second, the pro-inflammatory cytokines were cross-sectionally measured at the point of time where PD was initiated, which meant that we could not gather information about the trends of these inflammatory cytokines during PD therapy. However, we have taken into account many other variables, including age, levels of hemoglobin, existence of diabetes, alkaline phosphatase, preserved renal function, and dialysate adequacy. Our data has provided suitable predictive functions and clear illustrations about which types of PD patients initiating dialysis may have the worst outcomes.

The present study showed that high circulating levels of IL-18, IL-6, and IL-1ß could predict high all-cause mortality in PD patients. The mortality of PD patients was increased while they had increasing numbers of predictive factors including titers of IL-18 ≧ 804.3 pg/*ml*, titers of IL-6 ≧ 3.92 pg/*ml*, titers of IL-1ß ≧ 0.86 pg/*ml*, age ≧ 50 years-old, and diabetes. Higher level of IL-6 is associated with persistent decreasing levels of albumin and associated with higher mortality rate in PD patients. Future studies would focus both on early identification of those PD patients of high risk and to prospectively investigate how to lessen these risk factors.

## Conflicts of interest

The authors declare no conflicts of interest.
